# The Use of Angiotensin II for the Treatment of Post-cardiopulmonary Bypass Vasoplegia

**DOI:** 10.1007/s10557-020-07098-3

**Published:** 2020-10-21

**Authors:** Olga Papazisi, Meindert Palmen, A. H. Jan Danser

**Affiliations:** 1 Department of Cardiothoracic Surgery, Leiden University Medical Center, Leiden, The Netherlands; 2 Department of Internal Medicine, Division of Pharmacology, Erasmus Medical Center, Rotterdam, The Netherlands

**Keywords:** Angiotensin II, Vasoplegia, Cardiopulmonary bypass, Cardiac surgery

## Abstract

**Purpose:**

Vasoplegia is a common complication after cardiac surgery and is related to the use of cardiopulmonary bypass (CPB). Despite its association with increased morbidity and mortality, no consensus exists in terms of its treatment. In December 2017, angiotensin II (AII) was approved by the Food and Drug Administration (FDA) for use in vasodilatory shock; however, except for the ATHOS-3 trial, its use in vasoplegic patients that underwent cardiac surgery on CPB has mainly been reported in case reports. Thus, the aim of this review is to collect all the clinically relevant data and describe the pharmacologic mechanism, efficacy, and safety of this novel pharmacologic agent for the treatment of refractory vasoplegia in this population.

**Methods:**

Two independent reviewers performed a systematic search in PubMed, Embase, Web of Science, and Cochrane Library using relevant MeSH terms (Angiotensin II, Vasoplegia, Cardiopulmonary Bypass, Cardiac Surgical Procedures).

**Results:**

The literature search yielded 820 unique articles. In total, 9 studies were included. Of those, 2 were randomized clinical trials (RCTs) and 6 were case reports and 1 was a retrospective cohort study.

**Conclusions:**

AII appears to be a promising means of treatment for patients with post-operative vasoplegia. It is demonstrated to be effective in raising blood pressure, while no major adverse events have been reported. It remains uncertain whether this agent will be broadly available and whether it will be more advantageous in the clinical management of vasoplegia compared to other available vasopressors. For that reason, we should contain our eagerness and enthusiasm regarding its use until supplementary knowledge becomes available.

**Electronic supplementary material:**

The online version of this article (10.1007/s10557-020-07098-3) contains supplementary material, which is available to authorized users.

## Introduction

Vasoplegia is a complication that occurs in 5–25% of patients that undergo cardiac surgery on cardiopulmonary bypass (CPB) [1, 2]. It is defined by profound hypotension, low systemic vascular resistance (SVR), normal or increased cardiac output (CO), and a blunted or, sometimes, absent response to the administration of fluids and vasopressors [3]**.** Persistent hypotension leads to tissue hypoperfusion that might, ultimately, result in end-organ dysfunction. Therefore, vasoplegia is associated with increased perioperative morbidity and mortality [4–6]. Despite the fact that this syndrome is common in cardiac surgery patients and despite its poor prognosis, its underlying pathophysiological mechanism has yet to be elucidated and no consensus has been reached for a guided treatment strategy.

The current guidelines for the pharmacological treatment of vasoplegia include fluid resuscitation and catecholamine infusion, with norepinephrine (NE) being the first-line agent [7]. If those initial measures have failed to restore blood pressure (BP), other vasoactive substances might be included in the initial treatment regime. Second-line vasopressors might be other catecholamines, like phenylephrine, epinephrine, and dopamine, while other agents that can be used are arginine vasopressin (AVP), methylene blue, ascorbic acid, hydroxocobalamin, corticosteroids, and angiotensin II (AII). Although many different vasopressor drugs are available for the treatment of vasoplegia, significant inconsistency exists between countries and clinical centers in the management of vasodilatory shock [7] and yet, the mortality rate remains high [4].

Therefore, the aim of this systematic review is to investigate what is currently known about AII, a relatively new vasoactive substance, which was approved by the Food and Drug Administration (FDA) for use in vasodilatory shock in December 2017. The article focuses on the pharmacologic mechanism of AII and the clinically relevant data regarding its efficacy and safety from studies that have used AII in patients that underwent cardiac surgery on CPB.

### Study Selection

**Fig. 1:**
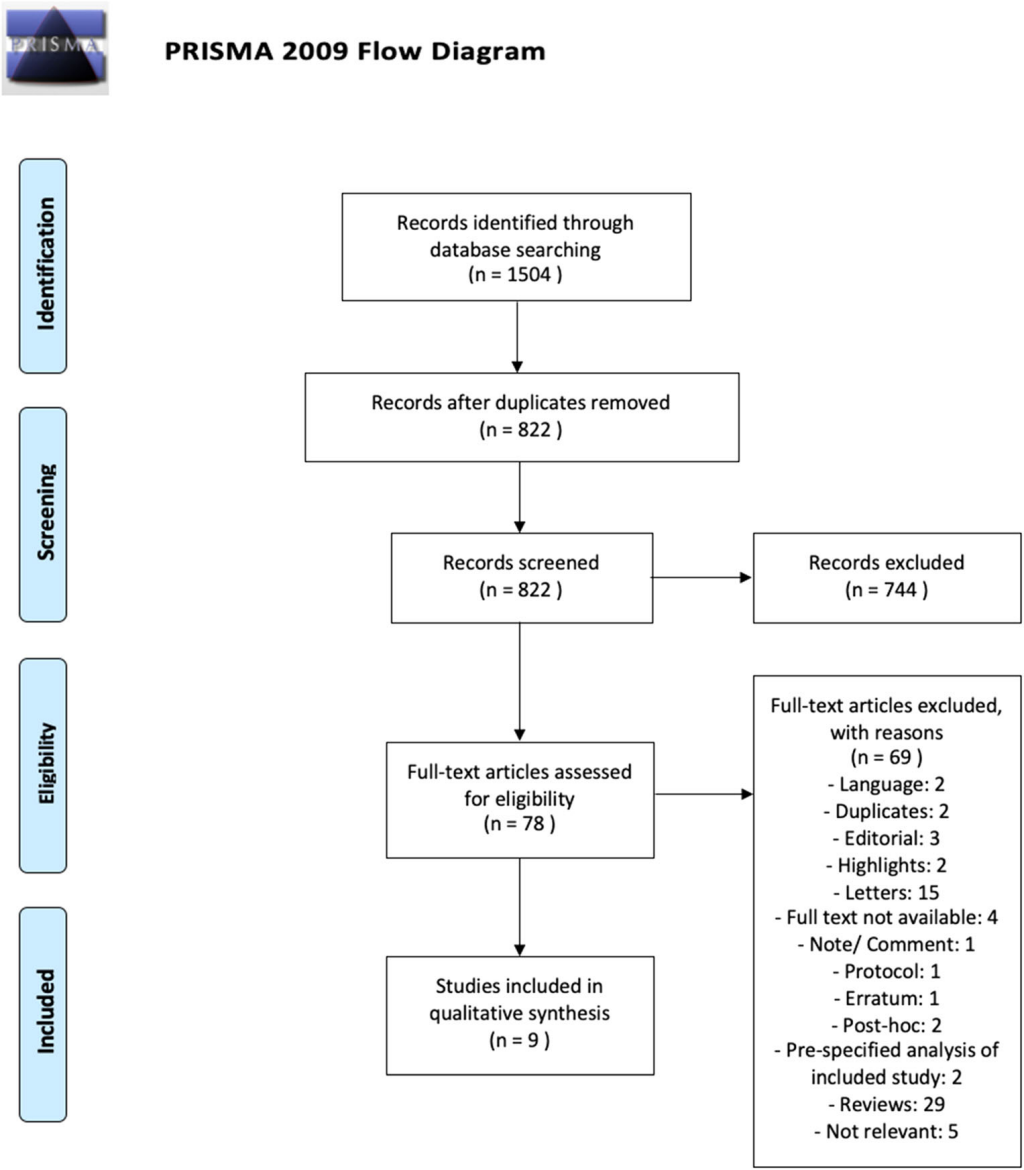
Flowchart of the systematic literature search and study selection procedure (RCTs, randomized clinical trials)

The search strategy initially yielded 820 unique, regular references. After the first screening, 76 studies were considered potentially relevant. The full text of those articles was then assessed and the reference lists of all eligible articles and reviews were manually checked for other relevant studies that were missed with the used search strategy. In total, 9 studies were included. Of those, 2 were randomized clinical trials (RCTs), 6 were case reports, and 1 was a retrospective cohort study (see Fig. 1, Appendix and Supplemental Table 1 and 2).

The extracted data did not allow us to perform a meta-analysis and therefore, we provide a narrative synthesis of the results. All findings are summarized in Table 1.

**Table 1:** Summary of all findings

	Type of article	Population	AII dose	Other vasopressors used	Findings
Geary et al. [8]	Case report	1	6 μg/min	PE NE	↑ MAP to 60 mmHg MAP =70–75 mmHg during post-CPB period
Thaker et al. [9]	Case report	2	6–7 μg/min	PE NE Epi	↑ PP rapidly
Bennett et al. [10]	RCT	AII: 10 vs PE: 10	2.5 mg /50 ml NS	PE	- One patient had to switch to AII due to lack of response to PE - Pre-operative HF was associated with ↑ need of vasoconstrictors - Renal function: X
Khanna et al. [11]	RCT	AII:163 vs placebo:158 (vasoplegia: 19; AII: 10 vs placebo: 9)	*Initiation:* 20 ng/kg/min *Between 3 h 15′–48 h:* 1.25–40 ng/kg/min		AII group:: - More patients met the primary end-point criteria (*P* < 0.001) - Tolerated greater decreases in AII doses and background vasopressors - Improvement in cardio vascular SOFA was greater (*P* = 0.01) - N/S differences in total SOFA score - N/S trend for lower 7- and 28-day mortality - Negative predictors: hypoalbuminemia, high vasopressor dose - Adverse events only in AII group (N/S): 1. Deep vein thrombosis 2. Tachycardia 3. Ventricular fibrillation
Evans et al. [12]	Case report	1	*Initiation:* 20 ng/kg/min *Titration:* 40 ng/kg/min	NE AVP MB Hydroxocobalamin	- ↑ MAP and SVR rapidly - ↓ NE dose
Wieruszewski et al. [13]	Case report	4	*Initiation*: 10–20 ng/kg/min *Titration:* 30 ng/kg/min	NE Epi AVP Ascorbic acid MB Hydroxocobalamin	- ↓ NE dose - ↑ MAP - Oliguria was improved - ↑ CVP: N/S
Wieruszewski et al. [14]	Case report	1	*Initiation:* 20 ng/kg/min	NE AVP Midodrine	- ↓ NE dose: from 0.12 μg/kg/min to 0.07 μg/kg/min - MAP ≥ 65 mmHg - Liver enzymes: X
Cutler et al. [15]	Case report	4	*Initiation:* 2.5–10 ng/kg/min *Titration:* 30–80 ng/kg/min	NE AVP Epi MB Hydroxocobalamin	- Variable responses - ↓ or discontinuation of background vasopressors
Wieruszewski et al. [16]	Retrospective study	270 (vasoplegia: 28)	*Initiation:* 20 ± 12 ng/kg/min *Titration:* 52 ± 24 ng/kg/min	NE Epi PE AVP Dopamine	- 67% classified as responders - ↑ MAP - ↓ NED - Lower lactate concentration and AVP use before AII initiation were associated with higher odds of hemodynamic responsiveness to AII - Responders exhibited a higher chance of 30-day survival

*Abbreviations: AI* angiotensin I, *AII* angiotensin II, *AEs* adverse events, *AKI* acute kidney injury, *ARDS* acute respiratory distress syndrome, *AVP* arginine vasopressin, *BP* blood pressure, *CV* cardiovascular, *CVP* central venous pressure, *Epi* epinephrine, *HF* heart failure, *MAP* mean arterial pressure, *MB* methylene blue, *NE* norepinephrine, *NED* norepinephrine equivalent dose, *NS* natural saline, *N/S* not significant, *PE* phenylephrine, *PP* perfusion pressure, *RCT* randomized clinical trial, *RRT* renal replacement therapy, *SAEs* serious adverse events, *VS* vasodilatory shock, *X* similar

### Pathophysiology of Vasoplegia

**Fig. 2:**
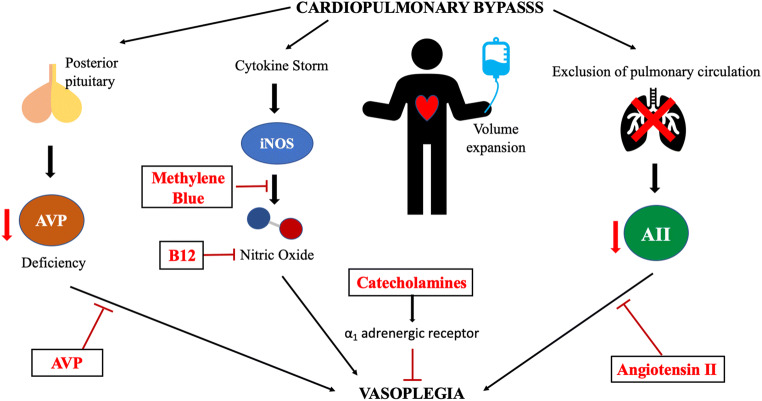
Pathophysiology of cardiopulmonary induced vasoplegia and existing therapies (AII, angiotensin II; AVP, arginine vasopressin; B12, hydroxocobalamin; iNOS, inducible nitric oxide synthase)

Vasoplegic syndrome (VS) is a complex, detrimental complication and even though its etiology remains unclear, many mechanisms are thought to be involved (Fig. 2). In fact, its pathophysiology is comparable to that of septic shock. The use of CPB in cardiac surgery is a generally accepted and extensively studied risk factor for the development of post-operative vasoplegia [17–19]. The disturbance of the homeostatic mechanisms during CPB results in cellular and neurohormonal alterations that play a role in the manifestation of VS. More specifically, the combination of surgical trauma and CPB induces changes in the vascular permeability, activates the coagulation and complement systems, and, as a result, triggers a systemic inflammatory response syndrome. The net results of this inflammatory response are thought to involve the inactivation of vasoconstrictor pathways and the concurrent activation of vasodilatory mechanisms, which in turn lead to hypotension, tissue hypoperfusion, and multiple organ dysfunction syndrome.

The use of CPB has been associated with an altered endothelial function and with increased production of NO [20, 21]. This aforementioned cytokine storm can enhance the expression of the inducible nitric oxide synthase (iNOS) in endothelial and vascular smooth muscle cells (VSMC). The activation of iNOS leads to an exaggerated production of nitric oxide (NO) from L-arginine, which, consecutively, results in vasodilation and additionally contributes to the resistance of the vasculature to vasopressor medication. Mainly, this is thought to be performed via the activation of myosin light-chain phosphatase and adenosine triphosphate (ATP)–sensitive potassium channels (K_ATP_) [22]. The activation of K_ATP_ induces a hyperpolarization which inhibits the normal function of calcium (Ca^2+^) channels causing vasodilation [23]. Thus, this mechanism might explain the blunted response of vasoplegic patients to the administration of catecholamines, which mediate vasoconstriction via the activation of those Ca^2+^ channels.

**Fig. 3:**
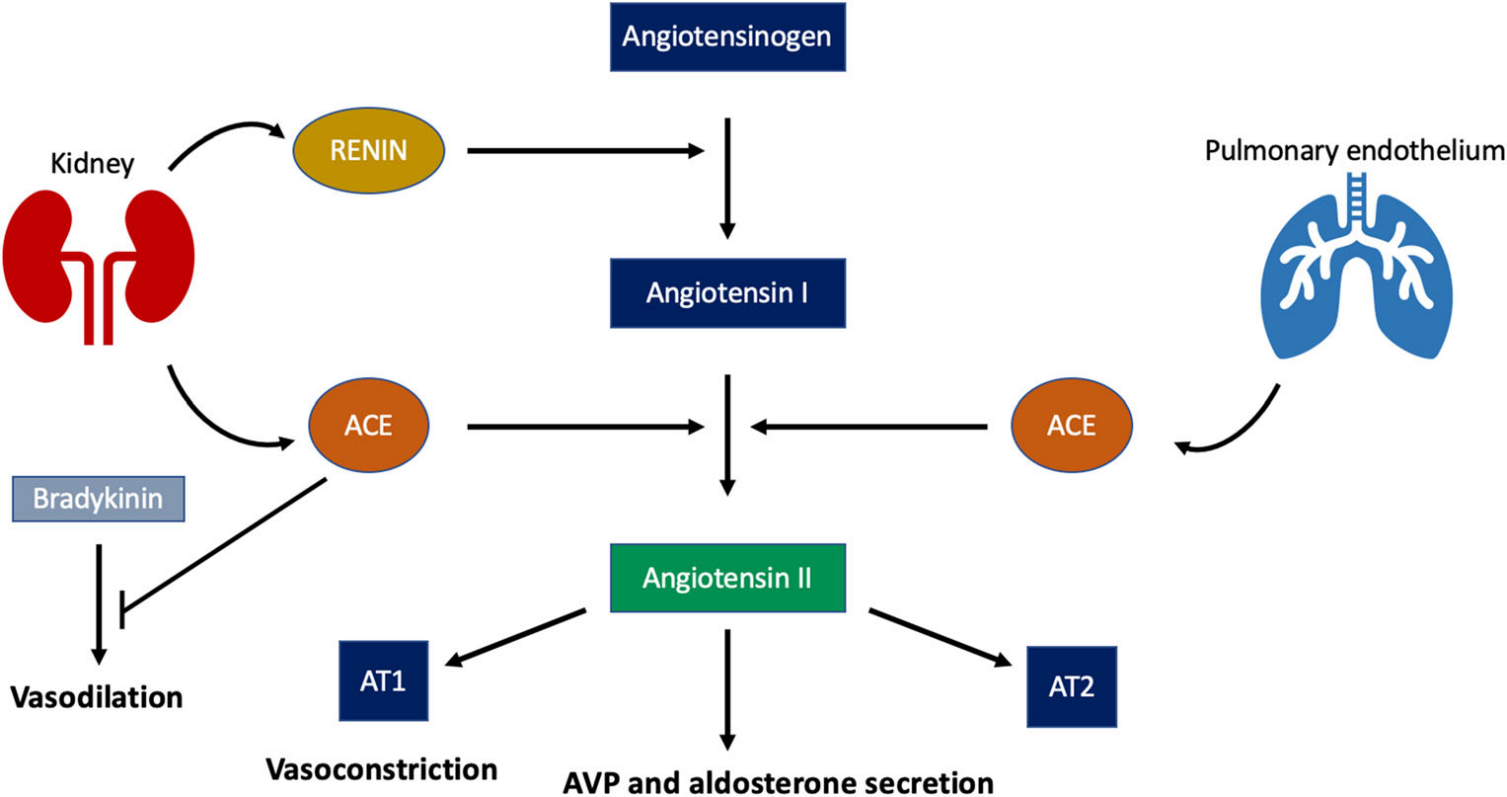
RAAS system (ACE, angiotensin-converting enzyme; AVP, arginine vasopressin; AT1, angiotensin receptor type 1; AT2, angiotensin receptor type 2)

Normally, a decrease in BP leads to the release of renin, thus activating the renin-angiotensin-aldosterone system (RAAS) axis (Fig. 3) [24]. Renin cleaves angiotensinogen to generate angiotensin I (AI) which is subsequently converted into angiotensin II (AII) by angiotensin-converting enzyme (ACE), an enzyme found on endothelial cell, particularly in the lungs. AII predominantly acts via AT1 receptors in order to increase vascular resistance and to enhance the secretion of both AVP from the posterior pituitary and aldosterone from the adrenal cortex. In addition, ACE is responsible for the degradation of bradykinin, a potent vasodilator. Thus, increased ACE activity would ultimately lead to an increase in BP. During cardiac surgery, the use of CPB bypasses the function of the lungs and, therefore, increases the concentration of bradykinin in blood [25]. In addition, the exclusion of the pulmonary circulation during CPB might also signify a hindered exposure of AI to pulmonary ACE and consequently, a diminished production of AII. ACE inhibitors (ACEi) have been widely studied and are currently recognized as an independent risk factor of post-operative vasoplegia [26–28]. This also supports the importance of AII in the maintenance of systemic vascular resistance (SVR).

Another component of this multifactorial condition, modulated by the RAAS axis, is AVP. AVP is a peptide hormone which is produced in the hypothalamus in response to baroreceptor and osmolar control [29]. The main biological functions of AVP are the regulation of water conservation and vascular constriction. The latter is mediated via V1 receptors that are located on the VSMC surface. Despite the fact that an increase in AVP levels would be expected in shock states, there have been reports of abnormally diminished AVP levels after cardiac surgery [30–32] that contribute to the development of hypotension and, consequently, to vasoplegia. The mechanisms that result in this AVP depletion have not been elucidated yet. However, Colson et al. [32] have described increased pre-operative levels of copeptin (a peptide derived from the C-terminus of the pre-pro-hormone of AVP, thus directly reflecting AVP exposure) and decreased post-operative AVP concentrations in vasoplegic patients, which might support the hypothesis that pre-operative stimulation of AVP production might lead to the depletion of its pituitary stores.

Taking into account the, at times, unsuccessful treatment of post-operative vasoplegia with conventional therapy and the physiological importance of AII in the maintenance of normal BP, considerable discussion arises regarding the safety and efficacy of this peptide in the correction of refractory hypotension after cardiac surgery on CPB.

### Angiotensin II

Angiotensin II was identified and recognized for its vasoconstrictor properties already in the 1940s. Some decades later, bovine AII was introduced (Hypertensin®, CIBA-Geigy Pharmaceuticals) for the treatment of vasodilatory shock. However, its availability was withdrawn in 2009 for reasons unrelated to the safety of the compound. In December 2017, a new form of AII was approved by the FDA (Giapreza®, La Jolla Pharmaceutical Company) which, unlike the chemical structure of the old pharmaceutical product, resembles the amino acid sequence of the human peptide. Giapreza® is intended for continuous intravenous administration with a recommended starting dosage at 20 ng/kg and must be diluted in 0.9% sodium chloride prior to use. The initial dose can be titrated to effect every 5 min and the maximum infusion should not exceed 80 ng/kg during the first 3 h. AII is metabolized in less than 1 min by aminopeptidase A and ACE-2 to angiotensin III and angiotensin-(1–7). The first metabolite constitutes 40% of the total AII vasoconstricting effect, while the latter opposes AII activity by exerting vasodilation. Last of all, AII metabolism is independent from renal and hepatic clearance and therefore, it should not be affected by renal or hepatic dysfunction.

### Efficacy and Safety

Despite the need for treatment alternatives of post-operative vasoplegia, our current knowledge and experience of this medication are limited and they mostly originate from circumstantial case reports. The first report to date the successful administration of AII in patients presenting with hypotension during cardiac surgery was in the 1960s [33]. More specifically, AII was reported to be a useful and safe medication for the increase of BP during mitral valve repair surgery for mitral stenosis, since it significantly decreased the time needed to perform the finger manipulation of the mitral valve. About 30 years later, some additional incidental reports were published that also supported the efficacy of AII in the treatment of refractory vasoplegia without resulting in any accompanying adverse effects (Table 1) [8, 9]. Those patients became hypotensive slightly after the initiation of CPB and they exhibited a blunted response to the administration of a_1_-adrenoceptor agonists. A mean AII dose of 6–7 μg/min was administered and a rapid increase in mean arterial blood pressure (MAP) was observed.

After the approval of the new form of AII, a few more case reports in cardiac surgery patients were published [12–14]. All of them described the successful management of post-operative, refractory VS with the addition of AII to the background vasopressors. In all cases but one, the initial dose of AII was 20 ng/kg/min. Only in one of the patients, who underwent bilateral orthotopic lung transplantation [13], a lower rate of AII (10 ng/kg/min) was initiated, in order to avoid a severe increase of the pulmonary vascular resistance. Nevertheless, the patient’s central venous pressure (CVP) did not rise significantly, while AII improved the patient’s oliguria. All patients exhibited immediate and fast hemodynamic responses to the administration of AII and this also allowed for a concomitant reduction in the background NE dose. In addition, Evans et al. [12] reported that despite the medical history of the patient in their case report with stage 3 chronic kidney disease, acute kidney injury (AKI) could be avoided, while simultaneously meeting the criteria of the Society of Thoracic Surgeons metric of preventing prolonged intubation. Lastly, despite the concerns of the physicians, no alterations were identified in another patient’s laboratory tests for liver function [14].

Similar were the findings of a relatively small randomized controlled clinical trial that was published in 2001 [10]. The investigators compared the efficacy of AII with phenylephrine in post-CPB patients who were taking ACEi. In total, 20 patients were included and randomized to the two treatment groups. The vasoconstrictor agent was administered if the MAP was less than 50 mmHg with an SVR index below 1500 dynes cm^5^ m^2^ and if pulmonary artery wedge pressure was more than 16 mmHg with a cardiac index being above 2.2 L min^−1^ m^−2^. The target MAP was 60–70 mmHg during CPB. Interestingly, one patient was unresponsive to the administration of phenylephrine and yet exhibited a MAP response to AII. Hence, the results from this study indicate that AII is a useful and safe alternative in the treatment of vasoplegia after cardiac surgery, especially when other vasoconstrictor agents have failed to correct the hypotension.

A recent case report described the use of AII in patients developing vasoplegia after heart transplant surgery [15]. This patient population is susceptible to post-operative vasoplegia and endures a great risk during the post-operative course [5]. Thus, their appropriate management is important. The authors noted a variable response to the administration of AII, with most of the patients exhibiting a favorable response which allowed a reduction or discontinuation of background vasopressors. No correlated side effects were noted by the use of AII and most importantly, none of the patients experienced any thrombotic episodes. Therefore, AII might be a valid option for therapy/management of post-operative hypotension in heart transplant surgery; however, whether all patients might benefit from this agent remains still a question.

Angiotensin II for the Treatment of High-Output Shock 3 (ATHOS-3) is the largest randomized, double-blind, controlled clinical trial so far, to investigate the use of AII in patients with vasodilatory shock [11]. Despite the fact that only 5.9% of the included patients had post-operative vasoplegia, this study still remains a major landmark for the determination of the clinical efficacy and safety of AII. The primary endpoint of ATHOS-3 was the response of MAP to treatment with an increase to equal or above 75 mmHg or an increase of 10 mmHg from baseline MAP. The secondary efficacy endpoints consisted of changes in the cardiovascular Sequential Organ Failure Assessment (SOFA) score and in the total SOFA score between baseline measurements and 48 h later. In addition, changes in heart rate, in catecholamine dose, and all-cause mortality were investigated. For this study, patients with refractory vasodilatory shock were included, for whom at least 25 mL/kg of fluids and high-dose vasoconstrictors were administered during the last 24 h. The ATHOS-3 investigators defined vasodilatory shock as a cardiac index of > 2.3 L/min/m^2^ or as central venous oxygen saturation of > 70% coupled with central venous pressure of > 8 mmHg, with a MAP between 55 and 70 mmHg. High-dose vasopressors were defined as norepinephrine or norepinephrine equivalent dose of another vasopressor (NED) > 0.2 μg/kg/min. In total, 321 patients were included, 163 of whom received AII and 158 received saline placebo as treatment. Most of the patients, however, were patients with sepsis (*N* = 259, 80%), while only 19 patients presented with vasoplegia. There were no significant differences between the two groups regarding their baseline group characteristics, the baseline MAP (median for all patients: 66 mmHg (interquartile range [IQR] 63.7–68.7 mmHg)), the APACHE II scores, the cardiac index (median for all patients: 3.1 L/min/m^2^ (IQR 2.6–3.8 L/min/m^2^)), and the exposure to ACE inhibitors. The investigators identified a significantly greater amount of patients from the AII group as having a successful MAP response at hour 3 after initiation of the study (69.9% vs. 23.4%, *P* < 0.001). Moreover, 67% of the patients from the AII group tolerated reductions in both AII doses and in other vasopressors, which led to a significant difference in the mean change in NED from baseline to hour 3 (AII: − 0.03 ± 0.10 μg/kg/min; placebo: 0.03 ± 0.23 μg/kg/min; *P* < 0.001). Concomitantly, the AII group demonstrated a significantly greater improvement of their cardiovascular SOFA score, even though no significant difference was found between the two different treatment groups regarding the total SOFA score. Lastly, no significant differences were reported between the two groups regarding the all-cause 7-day and 28-day mortality nor with regard to adverse events. It should be emphasized, however, that only patients from the AII group experienced deep vein thrombosis (1.8%) while none of the patients from the placebo treatment arm did. Therefore, the findings from this study indicate that AII is helpful and relatively safe in the management of vasodilatory shock. It was demonstrated that AII successfully increased BP in patients with refractory hypotension, it improved the cardiovascular SOFA scores, and, in addition, it permitted to lower the dose of background catecholamines.

In the ATHOS-3 protocol additional, pre-specified analyses were also planned [34–36]. First of all, differences in 28-day mortality were analyzed and compared between two subgroups of ATHOS-3 patients. The first group was characterized by the increased severity of illness (APACHE II > 30) and the second group by a MAP < 65 mmHg. The results from this study indicate that severely ill patients who additionally received AII demonstrated significantly lower 28-day mortality rates (*P* = 0.037). Yet, although the findings in patients with MAP < 65 mmHg showed a similar trend, no significance was reached in that group [36]. Ham et al. [34] investigated the differences in clinical outcomes between patients that received higher (> 5 ng/kg/min) and lower doses (≤ 5 ng/kg/min) of AII. They identified a group of patients with endogenous insufficiency of AII who proved to be more sensitive to the administration of AII. More specifically, significantly more patients from this group met the primary endpoint of the ATHOS-3 trial compared to patients receiving AII at rates > 5 ng/kg/min (89.9% vs 51.2%), which was also related to a reduction in the background vasopressor dependency. Moreover, total and cardiovascular SOFA scores were significantly better in patients that were highly sensitive to AII, and furthermore, those patients demonstrated a significantly higher survival rate at day 28 compared to the other group (67% vs 41%). Another pre-specified analysis focused on the association of plasma AI and AII levels and their ratio with clinical outcome [35]. The authors used the pre-quantified, baseline AI/AII ratios from the ATHOS-3 trial. Ratios were similar in the two treatment arms and their median was calculated to be 1.63. In addition, they measured the AI and AII levels in sera from 24 healthy volunteers. An interesting finding of this study was that vasodilatory shock patients had significantly higher AI levels and AI/AII ratios compared to healthy subjects (*P* < 0.0001), while AII levels were similar between the groups. Patients with ratios above 1.63 received greater NED at baseline. Not surprisingly, a significant correlation between ACE inhibitor exposure and higher AI/AII ratios (the well-known consequence of ACE inhibition) was also identified in this study. Lastly, a multivariate analysis performed in the placebo group of the ATHOS-3 trial revealed that the AI/AII ratio was a significant predictor of mortality (hazard ratio 0.54; *P* = 0.0111).

Post hoc analyses of the ATHOS-3 trial were also published. For instance, one of the post hoc analyses was performed in order to investigate the effect of AII treatment on clinical outcomes of patients with AKI treated with renal replacement therapy (RRT) at the initiation of the intervention [37]. This analysis showed that patients who received AII had a significantly greater survival rate at day 28 compared to patients who received placebo. In addition, a significantly higher proportion of patients who received AII were RRT- and ventilator-free at day 7. However, covariate-adjusted survival and ventilator dependency were not significantly better in any of the multivariate analysis models. The effect of AII was also investigated in a subgroup of patients who presented with acute respiratory distress syndrome (ARDS) [38]. A significantly higher proportion of ARDS patients that received AII met the primary endpoint of the ATHOS-3 trial, irrespective of the ARDS classification. Interestingly, the association between 28-day mortality rates and the increasing severity of ARDS was not as strong in the AII treatment arm as it was in the placebo group of patients. In another post hoc analysis, it was demonstrated that significantly more patients from the AII group exhibited a decrease in vasopressor requirements compared to the placebo group (65% vs 44%) and that this decrease was associated with a significant reduction in serious adverse events (SAEs) and, also, in AE resulting in death. A recently published post hoc analysis of the ATHOS-3 trial examined the correlation of plasma renin levels with the clinical outcome and attempted to identify a potential prognostic usefulness of this measurement [39]. The authors report that the baseline renin levels were 3 times higher than the upper limits of normal (ULN) in 76% of the patients of the ATHOS-3 trial. However, no differences were noted between the two different treatment arms. As expected, given that AII suppresses renin release, a positive correlation was found between the baseline renin levels and the baseline AI/AII ratio (*P* < 0.001) or between baseline renin and AI levels (*P* < 0.001) and the latter correlation remained constant also at hour 3 of treatment (*P* < 0.001). Patients from the AII treatment group exhibited a median reduction of 54.3% in renin levels compared with only 14.1% in the placebo group at hour 3 of treatment (*P* < 0.0001). Baseline renin levels did not influence the hemodynamic response to treatment in either group. However, a multivariate analysis showed that AII treatment in patients with increased baseline renin levels conferred protection from mortality (hazard ratio, 0.62; 95% Confidence Interval, 0.39–0.98; *P* = 0.0423). More specifically, those patients presented a significantly greater ICU discharge rate by day 28 (*P* = 0.02), a lower 28-day mortality rate (*P* = 0.0115), a greater RRT liberation rate by day 7 (*P* = 0.01), and a significantly greater change in cardiovascular SOFA score at 48 h. These favorable effects of AII, however, were not evident in patients with baseline renin concentrations below the population median. Interestingly, a multivariate analysis in the placebo group showed that, after adjusting for covariates, plasma renin levels represented an independent risk factor of mortality (*P* = 0.0013). Therefore, the results from this recent post hoc analysis suggest that the majority of patients with catecholamine-resistant vasodilatory shock suffer from a malfunctioned RAAS and, additionally, that plasma renin concentration measurements could develop into an innovative approach that will help physicians to detect patients who might benefit from AII infusions. Another post hoc analysis confirmed the cost-effectiveness of including Giapreza in the treatment of vasodilatory shock in ICUs of US hospital [40], while Anyanwu et al. [41] concluded that, specifically in AKI patients, even though the costs increase with the addition of AII in the standard vasopressor regime ($8657 (AII) vs $6517 (standard of care)), the increasing costs of RRT diminish the cost-difference between the two treatments.

Last of all, Klijian et al. [42] performed a post hoc analysis of the ATHOS-3 trial in order to examine the effect of AII in the subgroup of patients that had vasoplegia. In total, 19 patients that had post-operative vasoplegia were included in the ATHOS-3 trial (AII: 9 vs placebo: 10). Sixteen of those patients were included in the post hoc analysis (AII: 9 vs placebo: 7). A remarkably greater MAP response was noted in the AII group, since none of the patients in the placebo group met the primary endpoint of the study compared to almost 90% of the patients in the AII group that did. This greater hemodynamic response allowed for a rapid reduction in the AII dose already by 30 min after treatment initiation and also in the dose of background vasopressors at hours 3 and 12. On the contrary, patients from the placebo group did not experience any change in the dose of standard of care vasopressors at hour 3, while they even required an increase by 0.02 mg/kg/min at hour 12. Nevertheless, the 28-day mortality rates did not significantly differ between the two groups (AII: 10% vs placebo: 11%) and neither did the treatment-related adverse events.

For a better assessment of the efficacy and safety of AII administration in patients with vasodilatory shock, a retrospective study was conducted [16]. This study examined the effects of AII treatment in a total of 270 patients of which 28 presented with post-operative vasoplegia. The treatment population was categorized as AII responders (67%) and non-responders (33%), based on the achievement of a MAP > 65 mmHg combined with a stable or reduced total vasopressor dose. In line with previous findings, the hemodynamic response in the first group was characterized by a significantly greater increase in MAP (*P* < 0.001) and a concurrent significantly greater reduction in NED (*P* < 0.001). Furthermore, in contrast to ATHOS-3, a higher 30-day survival was noted in patients who responded to AII in the present study. In addition to the evaluation of AII efficacy, the authors assessed the existence of factors that could predict the patients’ response to AII. Lower lactate concentration and AVP administration before AII initiation were associated with a greater chance of hemodynamic responsiveness to AII. The association of higher lactate levels with a diminished responsiveness to AII might relate to a general disruption of the acid-base homeostasis in specific patients [43]; however, this still needs to be unraveled. On the other hand, the beneficial effect of AVP use on the responsiveness of patients to AII is in agreement with the well-known interaction between RAAS and AVP (Fig. 3) and the importance of those systems in the management of vasodilatory shock. As far as safety is concerned, this study identified less thromboembolic events (3%) and more events of thrombocytopenia and increased liver enzymes (24% each) compared to ATHOS-3, but none of those could be related to the use of AII.

## Discussion

AII is a novel vasopressor which was re-introduced into the market just a few years ago, for the clinical management of vasodilatory shock. The existing studies and case reports provide a positive impression of its usefulness and safety. More precisely, the use of AII was accompanied by rapid hemodynamic responses which, also, permitted minimization of catecholamine requirements. In addition to that, administration of AII seems to favor survival in patients with greater severity of illness and patients on RRT. Nonetheless, certainly, many questions regarding the use of AII in clinical practice remain unanswered.

For this new therapeutic option, more research is also needed in order to ascertain the existing risks. From the ATHOS-3 trial, it seemed that adverse events did not significantly differ between the two treatment arms; however, patients who received AII exhibited higher rates of deep vein thrombosis [11]. In the product information sheet, thromboembolism is included as a potential adverse event associated with AII use and therefore, its administration should be avoided in patients at risk of thromboembolic episodes.

It has still to be elucidated whether specific subgroups of patients might benefit from this new vasopressor drug. For instance, vasoplegia is a common complication in patients who undergo cardiac surgery on CPB and yet no golden standard exists regarding its treatment. Unfortunately, there is only limited data about the efficacy of AII in this patient population and these originate mostly from circumstantial case reports. Two randomized clinical trials [10, 11], one of which was the ATHOS-3 trial, also tested the use of AII in vasoplegic patients; however, the sample sizes are too small to generate concrete conclusions. Further investigation with large-scale randomized clinical trials is deemed necessary in order to justify the use and costs of this vasopressor in cardiac surgery patients.

Another interesting aspect of patients that present with post-operative vasoplegia is the presence of a malfunctioning ACE. The elimination of pulmonary circulation during CPB and the pre-operative use of ACEi might be accountable for the reduction of the endogenous production of AII in those patients. This endogenous AII deficiency seems to be associated with greater vasopressor needs and worst clinical outcomes [35], while it is also related to a more favorable response to the exogenous AII administration [34, 39]. Therefore, it is reasonable to consider that the baseline levels of AI, AII, and renin might represent a useful tool in the identification of patients that might actually benefit from the use of AII in the treatment of vasodilatory shock. Nevertheless, there is huge inter- and intra-individual variation in the levels of these components [44, 45] making the setting of cutoff values a major challenge.

In conclusion, AII appears to be a promising means of treatment for patients with post-operative vasoplegia. It is demonstrated to be effective in raising BP, while no major adverse events have been reported. It remains uncertain whether this agent will be broadly available and whether it will be more advantageous in the clinical management of vasoplegia compared to other available vasopressors. For that reason, we should contain our eagerness and enthusiasm regarding its use until supplementary knowledge becomes available.

## Electronic Supplementary Material

ESM 1 (DOCX 48 kb)

## Data Availability

PubMed, Embase, Web of Science, and Cochrane Library search.
