# Supplementary material for: The Use of Angiotensin II for the Treatment of Post-cardiopulmonary Bypass Vasoplegia
Source: Cardiovasc Drugs Ther. 2020 Oct 21;36(4):739–48. doi: 10.1007/s10557-020-07098-3 (PMC9270278; doi:10.1007/s10557-020-07098-3)
Supplement: Supplementary file 1 — (DOCX 48 kb) [file 10557_2020_7098_MOESM1_ESM.docx]

# Appendix

### Search strategy

This systematic review was based on Preferred Reporting Items for Systematic Reviews and Meta-analyses (PRISMA) Guidelines [1]. A comprehensive literature search was performed without language restrictions in four different databases (PubMed, Embase, Web of Science, Cochrane Library) using the following MeSH terms: vasoplegia, cardiopulmonary bypass, cardiac surgery and angiotensin II. For every concept, all relevant keyword variations were used, not only keyword variations in the controlled vocabularies of the various databases, but the free text word variations of these concepts as well. The search strategy was optimized for all consulted databases. The final search which was performed on March 2^nd^, 2020 yielded 820 studies. In order to keep this systematic review as up to date as possible, the search was rerun for possible new inclusions before publication.

### Study Eligibility

Clinical trials and case reports in which AII infusion was used for the treatment of vasoplegia in patients who underwent cardiac surgery on CPB were included. Only studies with patients ≥ 18 years were included. No linguistic limits were applied.

### Population, Intervention, Comparison, Outcome

Patient who developed vasoplegia after undergoing cardiac surgery on CPB and in whom AII was used as Intervention. The comparison included other vasopressor agents that were used and different dose-regimes between patients. Outcomes of interest were the clinical success rate expressed by the response of Mean Arterial Pressure (MAP), the mortality and the incidence of adverse effects (AEs).

### Study Selection

Two reviewers (OP and MP) have independently screened all the identified articles. The initial selection was conducted by only screening the titles and abstracts, while the final inclusion was decided upon reading the full text of the potentially relevant articles. Any disagreements regarding the final inclusion were settled by discussion. Relevant articles and reviews were manually checked for additional eligible studies or case reports.

### Data Collection and Analysis and Quality assessment

A predefined Google Spreadsheet form with audit trail was used to extract data from reports. Data collection included study and baseline characteristics of patients, indication for cardiac surgery, treatment characteristics and clinical outcomes. Two reviewers (OP and AHJD) independently assessed the extracted information for accuracy.

A single patient case report does not allow the estimation of an effect size and would only provide descriptive or narrative results.

Identified articles will be grouped into different categories based on the type of the study, e.g. randomized clinical trial (RCT) or case report. Consequently, these studies will be independently assessed for their quality by two different reviewers (OP and MP). An appropriate for each study Risk of Bias (RoB) tool will be used (Supplemental Table 1 and 2). Any disagreements will be recorded and resolved by involvement of a third reviewer (AHJD).

## Full search strategy

((("Angiotensin II"[mesh] OR "angiotensin II"[tw] OR "angiotensinII"[tw] OR "angiotensin 2"[tw] OR "angiotensin type 2"[tw] OR "angiotensin type II"[tw] OR "ANG II"[tw] OR "ANG 2"[tw] OR "ANG-(1-8)Octapeptide"[tw] OR "Angiotensin-(1-8) Octapeptide"[tw] OR "Angiotensin Amide"[tw] OR "Saralasin"[tw]) AND ("Vasoplegia"[Mesh] OR "vasoplegia"[tw] OR vasopleg*[tw] OR "Vasodilatory Shock"[tw] OR "high output shock"[tw] OR "Systemic Inflammatory Response Syndrome"[Mesh:NoExp] OR "SIRS"[tw] OR "systemic inflammatory response"[tw] OR "systemic inflammatory responses"[tw] OR "systematic inflammatory response"[tw] OR "systematic inflammatory responses"[tw] OR "Cytokine Release Syndrome"[Mesh] OR "Cytokine Release Syndrome"[tw] OR "Cytokine Release Syndromes"[tw] OR "cytokine storm"[tw] OR "cytokine storms"[tw])) OR (("Angiotensin II"[mesh] OR "angiotensin II"[tw] OR "angiotensinII"[tw] OR "angiotensin 2"[tw] OR "angiotensin type 2"[tw] OR "angiotensin type II"[tw] OR "ANG II"[tw] OR "ANG 2"[tw] OR "ANG-(1-8)Octapeptide"[tw] OR "Angiotensin-(1-8) Octapeptide"[tw] OR "Angiotensin Amide"[tw] OR "Saralasin"[tw] OR "Angiotensins"[Mesh] OR angiotensin*[tw] OR vasopressor*[tw] OR "Vasoconstrictor Agents"[Mesh] OR "Vasoconstrictor Agents"[Pharmacological Action]) AND ("Vasoplegia"[Mesh] OR "vasoplegia"[tw] OR vasopleg*[tw] OR "Vasodilatory Shock"[tw] OR "high output shock"[tw] OR "Systemic Inflammatory Response Syndrome"[Mesh:NoExp] OR "SIRS"[tw] OR "systemic inflammatory response"[tw] OR "systemic inflammatory responses"[tw] OR "systematic inflammatory response"[tw] OR "systematic inflammatory responses"[tw] OR "Cytokine Release Syndrome"[Mesh] OR "Cytokine Release Syndrome"[tw] OR "Cytokine Release Syndromes"[tw] OR "cytokine storm"[tw] OR "cytokine storms"[tw] OR "Hypotension"[majr] OR hypotens*[ti]) AND ("Cardiopulmonary Bypass"[Mesh] OR "cardiopulmonary bypass"[tw] OR cardiopulmonary bypass*[tw] OR "cardio-pulmonary bypass"[tw] OR cardio-pulmonary bypass*[tw] OR "postcardiopulmonary bypass"[tw] OR "Heart Lung Bypass"[tw] OR Heart Lung Bypass*[tw] OR "Cardiac Surgical Procedures"[mesh] OR "cardiac surgery"[tw] OR "Vascular Surgical Procedures"[mesh])) OR "Vasoplegia/drug therapy"[Majr] OR (("Angiotensins"[Mesh] OR angiotensin*[tw] OR "Angiotensin II"[mesh] OR "angiotensin II"[tw] OR "angiotensinII"[tw] OR "angiotensin 2"[tw] OR "angiotensin type 2"[tw] OR "angiotensin type II"[tw] OR "ANG II"[tw] OR "ANG 2"[tw] OR "ANG-(1-8)Octapeptide"[tw] OR "Angiotensin-(1-8) Octapeptide"[tw] OR "Angiotensin Amide"[tw] OR "Saralasin"[tw]) AND ("Cardiopulmonary Bypass"[Mesh] OR "cardiopulmonary bypass"[tw] OR cardiopulmonary bypass*[tw] OR "cardio-pulmonary bypass"[tw] OR cardio-pulmonary bypass*[tw] OR "postcardiopulmonary bypass"[tw] OR "Heart Lung Bypass"[tw] OR Heart Lung Bypass*[tw])))

**Supplemental Table 1.** Risk of bias assessment for Randomized Clinical Trials (RCTs)*.

| **RCT** | **A** | **B** | **C** | **D** | **E** | **F** | **G** |
| --- | --- | --- | --- | --- | --- | --- | --- |
| Khanna et al. [2] | **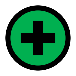** | **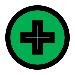** | **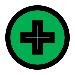** | **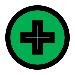** | **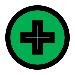** | **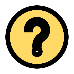** | **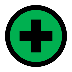** |
| Bennett et al. [3] | **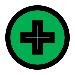** | **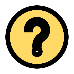** | **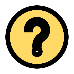** | **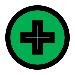** | **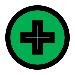** | **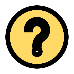** | **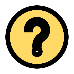** |

**
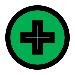
** Low risk of bias

**
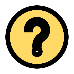
** Some concerns

**Cochrane risk-of-bias tool for randomized trials (RoB 2)*

A: Risk of bias arising from the randomization process

B: Risk of bias due to deviations from the intended interventions (effect of assignment to intervention)

C: Risk of bias due to deviations from the intended interventions (effect of adhering to intervention)

D: Missing outcome data

E: Risk of bias in measurement of the outcome

F: Risk of bias in selection of the reported result

G: Overall risk of bias

**Supplemental Table 2.** Risk of bias assessment for cohort studies*.

| Paper | Selection | | | | Comparability | Outcome | | | Total quality score |
| --- | --- | --- | --- | --- | --- | --- | --- | --- | --- |
|  | A | B | C | D | E | F | G | H |  |
| Wieruszewski et al.[4] |  | N/A |  |  |  |  |  |  | 7 |

** Newcastle-Ottawa Scale (NOS)*

A: Representativeness of the exposed cohort

B: Selection of the non-exposed cohort

C: Ascertainment of exposure

D: Demonstration that outcome of interest was not present at start of study

E: Comparability of cohorts on the basis of the design or analysis

F: Assessment of outcome

G: Follow-up was long enough for outcomes to occur

H: Adequacy of follow up of cohorts

**References**

1. Moher D, Liberati A, Tetzlaff J, Altman DG. Preferred reporting items for systematic reviews and meta-analyses: the PRISMA statement. PLoS Med. 2009;6:e1000097.

2. Khanna A, English SW, Wang XS, Ham K, Tumlin J, Szerlip H et al. Angiotensin II for the Treatment of Vasodilatory Shock. The New England journal of medicine. 2017;377:419-30.

3. Bennett SR, McKeown J, Drew P, Griffin S. Angiotensin in cardiac surgery: efficacy in patients on angiotensin converting enzyme inhibitors. European journal of heart failure. 2001;3:587-92.

4. Wieruszewski PM, Wittwer ED, Kashani KB, Brown DR, Butler SO, Clark AM et al. Angiotensin II Infusion for Shock: A Multicenter Study of Post-Marketing Use. Chest. 2020.
